# State-Specific Prevalence of Quit Attempts Among Adult Cigarette Smokers — United States, 2011–2017

**DOI:** 10.15585/mmwr.mm6828a1

**Published:** 2019-07-19

**Authors:** Kimp Walton, Teresa W. Wang, Gillian L. Schauer, Sean Hu, Henraya F. McGruder, Ahmed Jamal, Stephen Babb

**Affiliations:** ^1^Office on Smoking and Health, National Center for Chronic Disease Prevention and Health Promotion, CDC; ^2^McKing Consulting, Atlanta, Georgia; ^3^Department of Health Services, School of Public Health, University of Washington, Seattle, Washington.

From 1965 to 2017, the prevalence of cigarette smoking among U.S. adults aged ≥18 years decreased from 42.4% to 14.0%, in part because of increases in smoking cessation (*1**,**2*). Increasing smoking cessation can reduce smoking-related disease, death, and health care expenditures (*3*). Increases in cessation are driven in large part by increases in quit attempts (*4*). Healthy People 2020 objective 4.1 calls for increasing the proportion of U.S. adult cigarette smokers who made a past-year quit attempt to ≥80% (*5*). To assess state-specific trends in the prevalence of past-year quit attempts among adult cigarette smokers, CDC analyzed data from the 2011–2017 Behavioral Risk Factor Surveillance System (BRFSS) surveys for all 50 states, the District of Columbia (DC), Guam, and Puerto Rico. During 2011–2017, quit attempt prevalence increased in four states (Kansas, Louisiana, Virginia, and West Virginia), declined in two states (New York and Tennessee), and did not significantly change in the remaining 44 states, DC, and two territories. In 2017, the prevalence of past-year quit attempts ranged from 58.6% in Wisconsin to 72.3% in Guam, with a median of 65.4%. In 2017, older smokers were less likely than younger smokers to make a quit attempt in most states. Implementation of comprehensive state tobacco control programs and evidence-based tobacco control interventions, including barrier-free access to cessation treatments, can increase the number of smokers who make quit attempts and succeed in quitting (*2*,*3*).

BRFSS is an annual state-based telephone (landline and cellular) survey of a randomly selected representative sample of noninstitutionalized U.S. adults aged ≥18 years.* During 2011–2017, BRFSS sample sizes ranged from 441,456 (2014) to 506,467 (2011). Median survey response rates ranged from 45.3% (2017) to 53.0% (2011) for landlines and from 27.9% (2011) to 47.2% (2015) for cellular phones.

Overall and age group–specific (18–24, 25–44, 45–64, and ≥65 years) prevalences of smokers who made quit attempts were calculated for 2011–2017 for the 50 states, DC, Guam, and Puerto Rico. Making a past-year quit attempt was defined as answering yes to the question, “During the past 12 months, have you stopped smoking for 1 day or longer because you were trying to quit smoking?” Past-year quit attempts were assessed among both current cigarette smokers^†^ and former cigarette smokers who quit within the past year.^§^ Chi-square tests were performed to examine differences in past-year quit attempts between the years 2011 and 2017 (p<0.05). Logistic regression was used to assess overall changes in prevalence during 2011–2017, controlling for sex, age group, and race/ethnicity (p<0.05). Quartiles were mapped and assessed by U.S. Census region.^¶^ All analyses were conducted using SAS-callable SUDAAN software (version 11.0.3; RTI International) to account for the complex survey sampling design.

In 2017, the prevalence of past-year quit attempts ranged from 58.6% (Wisconsin) to 72.3% (Guam), with a median of 65.4% (North Carolina) (Table 1). The lowest quartile of quit attempt prevalence (58.6%–62.5%) included six states in the Midwest, four in the South, three in the West, and one in the Northeast (Figure). In comparison, in 2011, the prevalence of past-year quit attempts ranged from 57.4% (West Virginia) to 71.6% (New York), with a median of 64.9% (Mississippi). The prevalence of past-year quit attempts was significantly higher in 2017 compared with 2011 in four states (Alabama, Hawaii, Kansas, Louisiana) and one territory (Guam) and significantly lower in two states (New York and Wisconsin). During 2011–2017, past-year quit attempts increased in four states (Kansas, Louisiana, Virginia, and West Virginia, p-value for trend<0.05), and declined in two states (New York and Tennessee, p-value for trend<0.05).

**TABLE 1:** Percentage of current and former smokers aged ≥18 years who reported a past-year quit attempt,* by state/territory — Behavioral Risk Factor Surveillance System, United States, 2011–2017

State/Territory	% (95% CI)						
	2011	2012	2013	2014	2015	2016	2017
Alabama^†^	62.6 (59.3–65.8)	64.5 (61.3–67.8)	68.9 (65.2–72.5)	71.6 (68.6–74.5)	67.8 (64.5–71.1)	64.3 (60.8–67.7)	67.5 (64.1–70.9)
Alaska	65.4 (61.1–69.8)	65.5 (61.4–69.5)	65.8 (61.9–69.7)	65.6 (61.5–69.8)	68.0 (62.7–73.3)	64.0 (58.3–69.7)	63.6 (57.2–70.0)
Arizona	63.8 (58.3–69.3)	66.3 (62.2–70.3)	67.3 (61.7–72.9)	66.1 (62.9–69.3)	65.2 (61.2–69.2)	63.2 (59.3–67.0)	66.6 (64.2–69.0)
Arkansas	64.5 (60.2–68.8)	65.5 (61.9–69.2)	62.9 (59.0–66.8)	63.8 (59.6–68.1)	70.6 (66.1–75.1)	63.2 (58.2–68.2)	66.7 (61.6–71.8)
California	66.8 (64.4–69.3)	63.4 (60.4–66.4)	67.3 (64.1–70.4)	65.3 (61.9–68.8)	66.8 (63.9–69.7)	69.5 (66.6–72.3)	68.0 (64.2–71.7)
Colorado	67.0 (64.2–69.8)	66.2 (63.5–68.8)	64.0 (61.3–66.6)	70.3 (67.8–72.9)	69.1 (66.1–72.1)	67.8 (65.3–70.4)	68.2 (65.2–71.1)
Connecticut	68.2 (64.3–72.1)	70.6 (67.3–73.9)	72.5 (69.1–75.8)	68.0 (64.1–71.9)	66.6 (63.2–70.0)	70.5 (67.3–73.7)	71.6 (68.2–74.9)
Delaware	68.0 (63.9–72.1)	62.5 (58.4–66.7)	60.2 (55.9–64.6)	65.7 (60.8–70.6)	68.2 (63.5–73.0)	64.1 (59.3–68.9)	71.0 (66.5–75.4)
District of Columbia	69.6 (64.7–74.4)	74.8 (69.7–80.0)	74.4 (69.6–79.1)	71.7 (65.4–77.9)	76.0 (70.1–81.9)	71.4 (67.0–75.8)	70.5 (65.8–75.2)
Florida	68.4 (65.6–71.2)	71.4 (67.9–75.0)	69.0 (66.6–71.4)	72.5 (69.7–75.3)	71.3 (68.0–74.5)	69.6 (67.4–71.9)	67.6 (64.4–70.9)
Georgia	67.7 (64.6–70.7)	66.1 (62.3–69.9)	65.1 (61.7–68.5)	72.0 (68.4–75.5)	71.8 (67.8–75.8)	67.6 (63.7–71.4)	64.3 (60.5–68.1)
Hawaii^†^	60.7 (56.5–64.9)	66.7 (62.6–70.8)	70.3 (66.5–74.1)	67.7 (64.0–71.5)	68.0 (64.0–72.0)	66.7 (62.9–70.5)	67.0 (63.1–70.8)
Idaho	65.0 (60.4–69.6)	63.1 (57.5–68.6)	68.9 (64.8–73.0)	65.6 (61.2–70.0)	66.3 (62.1–70.6)	61.4 (56.4–66.3)	62.2 (57.3–67.1)
Illinois	65.6 (61.3–69.8)	68.2 (64.0–72.4)	64.2 (60.1–68.3)	65.7 (61.5–69.9)	68.6 (64.5–72.7)	67.0 (62.8–71.2)	64.8 (60.7–68.9)
Indiana	63.0 (60.2–65.9)	63.0 (60.3–65.7)	63.6 (60.9–66.2)	63.8 (61.1–66.4)	64.7 (60.6–68.8)	62.3 (59.5–65.2)	62.0 (59.6–64.3)
Iowa	60.6 (57.4–63.8)	64.1 (61.0–67.3)	59.8 (56.5–63.2)	63.0 (59.9–66.2)	62.4 (58.6–66.3)	59.2 (55.6–62.9)	59.9 (56.9–63.0)
Kansas^†,§^	61.1 (59.1–63.0)	63.0 (60.4–65.7)	63.6 (61.9–65.4)	65.6 (63.4–67.9)	62.7 (60.9–64.6)	63.1 (60.5–65.7)	64.3 (62.4–66.2)
Kentucky	58.2 (55.3–61.1)	59.6 (56.7–62.4)	56.2 (53.3–59.1)	61.6 (58.5–64.7)	58.7 (55.1–62.3)	59.1 (56.1–62.1)	62.1 (58.7–65.5)
Louisiana^†,§^	65.0 (62.1–67.9)	64.4 (61.0–67.8)	65.9 (61.3–70.5)	71.1 (68.4–73.8)	66.0 (62.1–69.9)	66.8 (62.3–71.3)	69.7 (66.3–73.1)
Maine	64.5 (61.9–67.0)	66.5 (63.8–69.1)	64.0 (60.7–67.3)	62.3 (58.9–65.6)	63.9 (60.4–67.4)	63.5 (60.0–66.9)	62.2 (58.5–66.0)
Maryland	61.8 (58.1–65.4)	66.7 (63.3–70.2)	67.6 (64.4–70.7)	67.3 (63.3–71.3)	66.0 (61.4–70.7)	67.4 (64.5–70.3)	65.9 (62.4–69.3)
Massachusetts	67.2 (64.7–69.7)	67.7 (65.3–70.0)	67.5 (64.6–70.4)	71.5 (68.6–74.4)	68.0 (64.7–71.3)	67.6 (63.9–71.2)	64.6 (59.8–69.3)
Michigan	65.5 (62.6–68.5)	68.5 (65.7–71.2)	68.0 (65.5–70.4)	66.9 (63.9–69.9)	67.9 (65.2–70.7)	64.7 (62.2–67.1)	66.2 (63.6–68.8)
Minnesota	64.3 (61.8–66.8)	64.5 (61.9–67.0)	68.3 (65.2–71.4)	67.2 (65.2–69.3)	65.1 (62.8–67.4)	64.3 (62.2–66.5)	63.8 (61.4–66.1)
Mississippi	64.9 (62.0–67.8)	66.0 (62.8–69.2)	69.4 (66.2–72.6)	68.2 (63.9–72.5)	72.1 (68.6–75.6)	67.7 (63.9–71.4)	61.1 (56.8–65.5)
Missouri	58.6 (55.2–62.1)	60.9 (57.5–64.4)	63.8 (60.3–67.3)	59.9 (56.2–63.6)	64.3 (60.7–67.8)	61.4 (57.6–65.3)	59.7 (56.2–63.1)
Montana	58.2 (55.1–61.3)	61.3 (58.3–64.4)	60.9 (57.9–63.8)	64.7 (61.1–68.3)	63.4 (59.3–67.5)	62.5 (58.4–66.5)	60.6 (56.6–64.7)
Nebraska	62.0 (60.1–63.9)	62.9 (60.7–65.1)	64.0 (61.3–66.8)	65.2 (62.8–67.6)	65.8 (63.2–68.5)	61.6 (58.5–64.6)	63.9 (61.0–66.9)
Nevada	58.4 (53.9–62.9)	66.6 (62.5–70.6)	62.5 (57.1–67.8)	71.5 (66.5–76.4)	72.0 (66.4–77.7)	63.2 (58.4–68.0)	62.7 (57.2–68.2)
New Hampshire	61.9 (58.0–65.7)	66.0 (62.0–70.1)	66.8 (63.1–70.5)	66.5 (62.4–70.7)	65.6 (61.5–69.6)	64.0 (59.3–68.6)	63.7 (58.8–68.6)
New Jersey	68.7 (66.1–71.3)	69.6 (67.0–72.1)	71.0 (68.3–73.7)	71.2 (68.3–74.1)	70.8 (67.4–74.1)	68.8 (64.5–73.1)	71.3 (67.7–75.0)
New Mexico	69.5 (66.8–72.3)	63.9 (60.9–66.8)	63.1 (59.9–66.3)	68.7 (65.2–72.1)	69.5 (65.6–73.4)	68.5 (64.4–72.6)	65.5 (61.8–69.3)
New York^†,¶^	71.6 (68.5–74.7)	73.1 (69.6–76.5)	70.5 (67.4–73.6)	70.2 (66.7–73.8)	70.2 (67.5–72.9)	67.4 (65.0–69.9)	66.4 (63.3–69.5)
North Carolina	66.9 (63.9–69.8)	68.4 (66.1–70.8)	65.1 (62.2–68.1)	66.3 (63.3–69.3)	68.0 (64.8–71.1)	67.7 (64.5–70.9)	65.4 (61.3–69.6)
North Dakota	59.6 (55.7–63.4)	59.2 (55.0–63.3)	58.7 (55.1–62.2)	63.0 (59.0–66.9)	64.2 (60.1–68.3)	61.0 (57.3–64.7)	62.2 (58.7–65.8)
Ohio^§^	61.2 (58.3–64.0)	61.9 (59.5–64.4)	65.9 (63.3–68.4)	67.4 (64.3–70.4)	65.0 (61.8–68.2)	63.4 (60.5–66.2)	61.7 (58.9–64.6)
Oklahoma	62.8 (59.8–65.7)	66.4 (63.6–69.2)	64.9 (62.1–67.7)	66.9 (64.0–69.9)	63.4 (59.7–67.1)	63.6 (60.0–67.2)	65.9 (62.5–69.2)
Oregon	65.4 (61.8–69.0)	69.9 (66.2–73.7)	65.1 (61.3–69.0)	66.9 (62.9–70.9)	64.6 (60.6–68.5)	66.4 (62.7–70.0)	62.5 (58.8–66.2)
Pennsylvania	65.8 (63.3–68.3)	66.2 (64.0–68.5)	66.7 (64.2–69.1)	65.4 (62.6–68.2)	67.1 (63.5–70.7)	65.3 (61.8–68.7)	64.3 (60.9–67.6)
Rhode Island	68.1 (64.6–71.6)	65.5 (61.3–69.6)	69.5 (65.8–73.2)	70.2 (66.0–74.4)	67.3 (62.8–71.7)	67.1 (62.6–71.5)	69.6 (64.9–74.3)
South Carolina	65.0 (62.1–67.8)	68.6 (66.0–71.1)	67.4 (64.7–70.1)	68.4 (65.7–71.1)	68.0 (65.2–70.9)	68.9 (66.1–71.7)	65.8 (62.9–68.7)
South Dakota	63.6 (59.3–68.0)	60.5 (57.0–63.9)	63.1 (59.1–67.2)	62.5 (58.2–66.9)	64.1 (59.5–68.6)	62.5 (57.3–67.7)	64.5 (59.4–69.6)
Tennessee^¶^	66.2 (61.1–71.4)	66.6 (63.5–69.8)	66.8 (63.3–70.4)	64.3 (60.1–68.4)	62.1 (58.1–66.0)	65.0 (61.3–68.6)	60.3 (56.6–64.0)
Texas	69.4 (66.6–72.3)	67.3 (64.2–70.4)	69.9 (67.0–72.9)	72.2 (69.3–75.0)	67.6 (64.2–70.9)	71.1 (67.5–74.6)	70.7 (66.6–74.7)
Utah	70.0 (66.8–73.1)	71.7 (68.5–74.9)	69.5 (66.5–72.6)	71.1 (68.3–73.8)	65.5 (62.0–69.1)	69.3 (65.5–73.2)	66.4 (62.8–70.1)
Vermont	62.7 (59.0–66.5)	69.3 (65.6–72.9)	63.9 (60.1–67.6)	66.0 (62.6–69.4)	63.6 (59.9–67.4)	58.2 (54.1–62.4)	66.0 (62.1–70.0)
Virginia^§^	63.9 (60.2–67.7)	65.4 (62.1–68.8)	65.8 (62.8–68.8)	66.4 (63.5–69.4)	69.0 (65.8–72.1)	67.8 (64.9–70.8)	66.4 (63.2–69.5)
Washington	64.7 (61.4–68.0)	65.8 (63.3–68.3)	67.5 (64.7–70.3)	68.9 (65.7–72.0)	65.0 (62.3–67.7)	63.7 (61.0–66.4)	68.1 (65.4–70.8)
West Virginia^§^	57.4 (54.1–60.6)	56.1 (53.0–59.1)	59.7 (56.8–62.7)	59.1 (56.1–62.0)	60.5 (57.5–63.4)	60.8 (58.1–63.5)	61.6 (58.5–64.8)
Wisconsin^†^	67.0 (62.9–71.1)	68.3 (64.2–72.3)	71.3 (67.6–75.1)	66.3 (62.5–70.2)	66.8 (63.0–70.7)	67.6 (63.6–71.6)	58.6 (54.3–62.8)
Wyoming	61.3 (57.9–64.7)	61.6 (56.9–66.3)	62.7 (58.8–66.6)	63.0 (58.2–67.7)	63.3 (58.7–67.9)	60.3 (55.2–65.5)	65.0 (60.9–69.0)
Guam^†^	63.5 (58.5–68.6)	71.9 (66.8–76.9)	76.4 (71.6–81.2)	74.0 (69.1–78.9)	70.3 (63.9–76.7)	69.1 (62.0–76.3)	72.3 (66.6–77.9)
Puerto Rico	66.0 (62.1–70.0)	70.5 (66.5–74.4)	76.4 (72.1–80.8)	72.4 (68.1–76.7)	73.8 (68.9–78.6)	74.4 (69.7–79.1)	67.1 (61.5–72.7)
**Median**	**64.9**	**66.1**	**65.9**	**66.9**	**66.8**	**65.0**	**65.4**

**Abbreviation:** CI = confidence interval.

* Quit attempt percentages were calculated among current cigarette smokers who answered yes to the question “During the past 12 months, have you stopped smoking for 1 day or longer because you were trying to quit smoking?” and also among former cigarette smokers who answered “within the past month,” “within the past 3 months,” “within the past 6 months,” or “within the past year” to the question “How long has it been since you last smoked a cigarette, even one or two puffs?”

^†^ Statistically significant difference (p<0.05) between 2011 and 2017.

^§^ Statistically significant increasing linear trend during 2011–2017.

^¶^ Statistically significant decreasing linear trend during 2011–2017.

**FIGURE:**
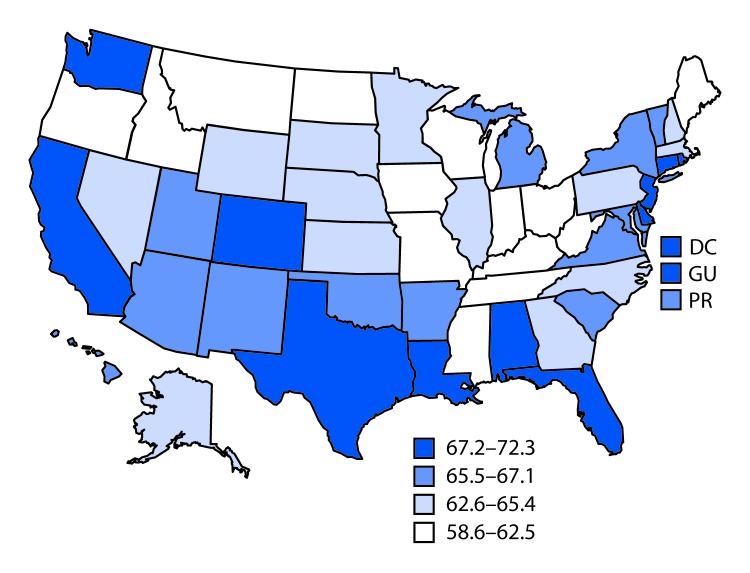
Percentage of current and former cigarette smokers aged ≥18 years who reported a past-year quit attempt* — Behavioral Risk Factor Surveillance System, United States, 2017^†^ **Abbreviations:** DC = District of Columbia; GU = Guam; PR = Puerto Rico. * Quit attempt percentages were calculated among current cigarette smokers who answered yes to the question “During the past 12 months, have you stopped smoking for 1 day or longer because you were trying to quit smoking?” and also among former cigarette smokers who answered “within the past month,” “within the past 3 months,” “within the past 6 months,” or “within the past year” to the question “How long has it been since you last smoked a cigarette, even one or two puffs?” ^†^ Median = 65.4%.

In 2017, the prevalence of past-year quit attempts generally decreased with increasing age (Table 2). The median prevalence of past-year quit attempts was 76.4% among persons aged 18–24 years (Hawaii), 68.6% among persons aged 25–44 years (Kansas), 60.8% among persons aged 45–64 years (Illinois), and 55.8% among persons aged ≥65 years (DC) (Table 2).

**TABLE 2:** Percentage of current and former cigarette smokers aged ≥18 years who reported a past-year quit attempt,* by state/territory and age group — Behavioral Risk Factor Surveillance System, United States, 2017

State/Territory	% (95% CI)			
	18–24 yrs	25–44 yrs	45–64 yrs	≥65 yrs
Alabama	79.3 (66.9–87.9)	69.5 (63.3–75.1)	64.2 (59.1–69.0)	57.7 (50.1–65.0)
Alaska	70.8 (45.9–87.3)	60.1 (49.4–70.0)	66.9 (58.3–74.4)	58.5 (45.6–70.4)
Arizona	78.8 (69.7–85.8)	74.1 (69.9–77.9)	59.7 (56.1–63.2)	52.6 (47.5–57.6)
Arkansas	72.7 (46.9–88.9)	73.8 (65.1–81.0)	60.5 (53.2–67.3)	50.1 (40.9–59.3)
California	78.4 (68.2–86.0)	65.1 (59.0–70.8)	70.1 (64.0–75.7)	66.3 (55.6–75.5)
Colorado	70.7 (60.2–79.3)	74.0 (69.4–78.1)	63.2 (58.2–68.0)	52.3 (45.2–59.4)
Connecticut	76.7 (62.8–86.6)	79.0 (73.2–83.9)	65.3 (60.2–70.0)	58.7 (50.0–66.8)
Delaware	84.5 (69.7–92.8)	77.4 (69.9–83.5)	63.1 (56.0–69.7)	55.9 (43.5–67.6)
District of Columbia	78.1 (53.9–91.6)	65.7 (57.2–73.3)	74.9 (68.6–80.2)	71.2 (61.4–79.3)
Florida	85.5 (75.5–91.8)	69.9 (63.8–75.3)	65.8 (60.7–70.5)	57.4 (49.3–65.1)
Georgia	74.1 (58.0–85.5)	67.1 (60.6–72.9)	61.0 (55.0–66.6)	55.4 (47.1–63.5)
Hawaii	76.4 (61.2–87.0)	70.7 (64.7–76.0)	62.1 (56.0–67.9)	50.0 (39.0–61.1)
Idaho	78.9 (64.5–88.5)	64.8 (56.3–72.4)	55.3 (47.1–63.3)	55.5 (44.6–66.0)
Illinois	60.5 (40.2–77.7)	70.1 (63.5–75.9)	60.8 (54.6–66.6)	60.3 (51.4–68.7)
Indiana	71.4 (62.1–79.2)	62.6 (58.4–66.5)	60.4 (57.1–63.7)	52.9 (47.7–58.1)
Iowa	63.6 (50.5–75.0)	63.5 (58.4–68.2)	56.1 (51.6–60.6)	55.1 (47.7–62.4)
Kansas	76.8 (70.2–82.3)	68.6 (65.5–71.6)	57.5 (54.5–60.5)	52.5 (47.8–57.2)
Kentucky	72.3 (56.9–83.7)	67.7 (62.4–72.6)	53.0 (47.5–58.3)	62.2 (53.9–69.8)
Louisiana	68.1 (55.1–78.8)	74.4 (69.0–79.2)	65.5 (59.9–70.7)	64.2 (54.7–72.7)
Maine	80.8 (57.8–92.8)	62.5 (56.2–68.4)	59.8 (54.5–65.0)	54.3 (46.6–61.8)
Maryland	65.5 (49.1–78.8)	69.5 (63.3–75.0)	62.0 (57.2–66.6)	65.0 (57.8–71.5)
Massachusetts	70.3 (50.9–84.5)	70.0 (61.6–77.3)	59.4 (51.9–66.5)	59.1 (46.6–70.6)
Michigan	76.6 (66.4–84.4)	67.2 (62.7–71.5)	64.1 (60.2–67.8)	61.3 (54.8–67.4)
Minnesota	71.5 (62.0–79.5)	67.2 (63.2–70.9)	60.0 (56.4–63.4)	55.8 (50.1–61.3)
Mississippi	85.7 (67.1–94.6)	56.2 (48.2–63.9)	62.5 (56.5–68.0)	53.5 (44.7–62.0)
Missouri	71.4 (60.0–80.6)	59.4 (53.3–65.3)	57.8 (52.6–62.9)	52.8 (45.0–60.5)
Montana	60.7 (45.0–74.4)	66.2 (59.8–72.1)	57.8 (51.3–64.1)	48.9 (40.1–57.7)
Nebraska	75.1 (65.2–82.9)	68.6 (63.8–73.0)	57.1 (52.4–61.7)	51.2 (43.9–58.4)
Nevada	76.3 (52.4–90.4)	67.0 (57.5–75.3)	60.9 (51.6–69.5)	51.2 (40.1–62.2)
New Hampshire	72.9 (53.7–86.1)	61.8 (52.8–70.0)	62.3 (56.1–68.1)	63.6 (54.5–71.7)
New Jersey	77.9 (57.7–90.1)	76.0 (69.7–81.3)	64.9 (59.4–70.0)	69.2 (60.9–76.5)
New Mexico	69.9 (54.5–81.8)	70.0 (63.5–75.7)	64.3 (58.3–69.8)	51.5 (43.4–59.4)
New York	70.8 (55.8–82.4)	68.9 (63.9–73.6)	64.4 (59.7–68.8)	59.9 (52.2–67.1)
North Carolina	68.2 (53.4–80.1)	71.3 (64.1–77.6)	58.6 (51.8–65.0)	63.8 (53.0–73.3)
North Dakota	84.4 (72.3–91.8)	67.8 (61.7–73.3)	47.4 (42.2–52.6)	54.6 (47.6–61.5)
Ohio	73.3 (61.7–82.4)	63.4 (58.5–68.0)	58.8 (54.5–62.9)	53.8 (47.5–60.1)
Oklahoma	75.0 (62.7–84.2)	68.0 (62.3–73.1)	63.3 (58.0–68.2)	52.8 (45.6–59.8)
Oregon	58.9 (43.7–72.6)	69.2 (63.3–74.6)	57.9 (51.9–63.7)	55.4 (45.7–64.7)
Pennsylvania	80.6 (69.7–88.2)	66.6 (60.7–72.0)	58.9 (53.8–63.9)	57.6 (48.0–66.6)
Rhode Island	80.8 (59.0–92.5)	68.7 (59.5–76.6)	68.4 (62.2–74.0)	65.8 (56.4–74.1)
South Carolina	72.5 (60.0–82.3)	69.1 (64.0–73.8)	63.1 (58.8–67.3)	55.8 (49.5–61.8)
South Dakota	76.9 (61.2–87.5)	68.7 (59.4–76.7)	58.0 (50.2–65.5)	52.0 (41.3–62.5)
Tennessee	77.8 (64.5–87.1)	62.9 (56.5–68.8)	55.5 (50.0–60.9)	49.1 (40.8–57.5)
Texas	78.0 (62.1–88.5)	70.5 (64.2–76.1)	70.6 (63.5–76.8)	59.7 (47.2–71.1)
Utah	76.9 (65.7–85.3)	69.6 (64.1–74.7)	58.4 (51.8–64.7)	56.4 (44.6–67.6)
Vermont	86.5 (72.0–94.1)	64.3 (57.3–70.7)	62.4 (56.2–68.3)	60.3 (51.2–68.8)
Virginia	80.4 (68.4–88.6)	71.6 (66.3–76.3)	59.0 (53.9–64.0)	53.6 (46.2–60.8)
Washington	77.3 (66.0–85.7)	72.4 (68.1–76.4)	61.6 (57.1–65.8)	60.3 (53.6–66.7)
West Virginia	84.7 (73.5–91.7)	62.0 (56.6–67.0)	56.4 (51.9–60.8)	50.4 (43.2–57.5)
Wisconsin	66.4 (49.2–80.2)	58.8 (51.7–65.6)	56.3 (49.9–62.5)	57.7 (47.2–67.6)
Wyoming	73.7 (60.6–83.6)	67.4 (60.6–73.6)	59.5 (52.9–65.7)	57.5 (48.5–66.0)
Guam	96.0 (87.8–98.8)	71.8 (62.0–80.0)	64.9 (55.3–73.5)	61.8 (37.6–81.2)
Puerto Rico	87.3 (73.0–94.6)	75.2 (66.3–82.4)	53.6 (43.9–63.0)	54.3 (39.0–68.7)
**Median**	**76.4**	**68.6**	**60.8**	**55.8**

**Abbreviation:** CI = confidence interval.

* Quit attempt percentages were calculated among current cigarette smokers who answered yes to the question “During the past 12 months, have you stopped smoking for 1 day or longer because you were trying to quit smoking?” and also among former cigarette smokers who answered “within the past month,” “within the past 3 months,” “within the past 6 months,” or “within the past year” to the question “How long has it been since you last smoked a cigarette, even one or two puffs?”

## Discussion

Among adult smokers in 2017, approximately 60%–70% had made a quit attempt in the past year, with variations in prevalences observed among states and territories. However, no state or territory met the national Healthy People 2020 objective 4.1 target of 80% (*5*). Moreover, only four states and one territory had a significantly higher prevalence of quit attempts in 2017 than in 2011, and only four states experienced a significant increase in quit attempt prevalence during this period. Most states experienced no change in quit attempt prevalence during 2011–2017. Finally, in 2017, past-year quit attempts generally decreased as respondent age increased across states and territories. The limited progress in increasing quit attempts reported in this study, together with the variation in quit attempt prevalence among states, underscores the importance of enhanced efforts to motivate and help smokers to quit.

A previous study, using 2001–2013 BRFSS data, found that the prevalence of past-year quit attempts among adult cigarette smokers increased significantly in 29 states and one territory during 2001–2010 and increased in one state and one territory while decreasing in one state during 2011–2013 (*6*). Another study that examined state-specific quit attempt prevalence by insurance status using 2014 BRFSS data found that, overall, adult smokers enrolled in Medicaid were more likely to make a past-year quit attempt than privately insured and uninsured smokers, although wide variations were observed in state-specific quit attempt prevalence (*7*).

The population quit rate is driven by two factors: prevalence of quit attempts and prevalence of successful quitting among smokers who make a quit attempt (*4*). Accordingly, increasing quit attempts is an important strategy to increase the population quit rate (*4*). CDC has identified increasing quit attempts as an important goal for state and national tobacco control efforts (*3*). Because most smokers make multiple quit attempts before succeeding, as many as 30 on average (*8*), tobacco dependence is viewed as a chronic, relapsing condition that requires repeated intervention (*9*). Smokers should be encouraged to keep trying to quit until they succeed, and health care providers should be encouraged to keep supporting smokers until they quit (*9*). Both smokers and providers can be reminded that, despite the barriers to quitting, three of five U.S. adults who ever smoked have quit successfully (*10*). In addition, providers and media campaigns can inform smokers that quitting is beneficial at any age, and that it is never too late to quit (*3*).

Proven tobacco control interventions, including tobacco price increases, comprehensive smoke-free laws, high-impact antitobacco mass media campaigns that promote free cessation resources like state quitlines, and barrier-free access to evidence-based cessation treatments, can work together to prompt smokers to make quit attempts and to give them a better chance of quitting successfully (*2*,*3*). Increases in quit attempts and successful cessation are also driven by comprehensive state cessation efforts, which include activities to 1) promote health systems change to integrate tobacco dependence treatment into routine clinical care; 2) improve cessation insurance coverage and increase use of covered cessation treatments; and 3) increase the reach and impact of state quitlines (*3*). Variations in the prevalence of smokers’ quit attempts among states might reflect, in part, differences in the extent to which states have implemented these interventions.

The findings in this report are subject to at least four limitations. First, these findings might not be generalizable to the entire U.S. population because the survey design excluded persons who reside in institutional settings. Second, adults without cellular or landline telephone service are excluded from BRFSS surveys. Third, these data are self-reported, and are therefore subject to recall and social desirability biases, which might affect results overall and which might differ among states. Finally, BRFSS response rates vary by state; even after adjusting for nonresponse, low response rates can increase the potential for bias if there are systematic differences between respondents and nonrespondents.

The variation in quit attempt prevalences among states described in this report suggests that states have an opportunity to further increase the prevalence of quit attempts. Increased implementation of proven tobacco control interventions (e.g., tobacco price increases, smoke-free policies, media campaigns, and barrier-free access to cessation treatments) can increase the number of smokers who make a quit attempt and who succeed in quitting (*2**,**3*). Implementation of these interventions might also reduce the variation in quit attempt prevalences among states observed in this study. Increasing quit attempts among adult smokers can help drive increases in smoking cessation. In addition, it is important to continue tracking cessation behaviors, including quit attempts, among states and territories to monitor future trends in these behaviors.

**Summary**

**What is already known about this topic?**

Increasing the prevalence of quit attempts and successful quitting is important to increase smoking cessation and to reduce smoking-related disease, death, and costs.

**What is added by this report?**

In 2017, at least six in 10 adult smokers reported trying to quit in the past year in almost all states. In that year, the prevalence of past-year quit attempts ranged from 58.6% (Wisconsin) to 72.3% (Guam), with a state/territory median of 65.4%. During 2011–2017, quit attempt prevalence increased in four states and decreased in two states; quit attempt prevalence did not change significantly in the remaining 44 states, DC, and two territories over this period.

**What are the implications for public health practice?**

Increased implementation of proven tobacco control interventions, such as tobacco price increases, smoke-free policies, mass media campaigns, and barrier-free access to evidence-based cessation treatments, can increase the number of smokers who make a quit attempt and who succeed in quitting.

## Footnotes

*
https://www.cdc.gov/brfss .

† Current cigarette smokers were defined as persons aged ≥18 years who reported smoking at least 100 cigarettes in their lifetime and smoking “every day” or “some days” at the time of the survey.

§ Former cigarette smokers were defined as persons aged ≥18 years who reported smoking at least 100 cigarettes in their lifetime and who do not smoke now. Quitting within the past year was defined as answering “within the past month,” “within the past 3 months,” “within the past 6 months,” or “within the past year” to the question “How long has it been since you last smoked a cigarette, even one or two puffs?”

¶ *Northeast:* Connecticut, Maine, Massachusetts, New Hampshire, New Jersey, New York, Pennsylvania, Rhode Island, and Vermont. *Midwest:* Illinois, Indiana, Iowa, Kansas, Michigan, Minnesota, Missouri, Nebraska, North Dakota, Ohio, South Dakota, and Wisconsin. *South:* Alabama, Arkansas, Delaware, District of Columbia, Florida, Georgia, Kentucky, Louisiana, Maryland, Mississippi, North Carolina, Oklahoma, South Carolina, Tennessee, Texas, Virginia, and West Virginia. *West:* Alaska, Arizona, California, Colorado, Hawaii, Idaho, Montana, Nevada, New Mexico, Oregon, Utah, Washington, and Wyoming.
